# Mechanistic or Relational Worldview for Talent Identification Research in Sport Science? Both—But With a Preference!

**DOI:** 10.17505/jpor.2023.25813

**Published:** 2023-12-07

**Authors:** Bryan Charbonnet, Achim Conzelmann

**Affiliations:** Institute of Sport Science, Department of Sport Psychology and Research Methods, University of Bern, Bern, Switzerland

**Keywords:** talent selection, diagnosis, prediction, variable-oriented, person-oriented, developmental science

## Abstract

This paper situates talent identification research in sport science within the broader context of developmental science, offering a conceptual framework informed by two (meta-)theoretical worldviews: the Cartesian-split-mechanistic and processual-relational worldviews. Although these worldviews are not explicitly named in the field of talent identification research, we demonstrate their implicit adoption through theoretical and methodological discourse. After comparing applications, benefits, and limitations of each worldview, we briefly discuss whether their bodies of knowledge are incompatible, competitive, or complementary. We suggest each worldview provides complementary insights with a penchant for generating nomothetic and group-specific and type-specific and idiographic knowledge, respectively.

## Introduction

The problem of talent identification – “diagnosing each individual, and steering him toward his fittest place” (Hall, 1917, p. 11) – is a classical, long-lasting societal problem in various domains (e.g., education, music). Some researchers defined it as “the supreme problem” (Hall, 1917, p. 11; Ployhart et al., 2017, p. 291). One domain where talent identification holds particular relevance is in sport, because professional teams and national federations have a vested interest in selecting top athletes to compete for global recognition (e.g., Olympic medals).

In the early days of talent identification research in sport, a narrow, static solution prevailed: basing talent identification on one sport-specific performance measurement at one measurement point (Abbott & Collins, 2002; Hohmann, 2009). Over time, the theoretical landscape shifted from a narrow, static solution to a *broad, dynamic* one (Hohmann, 2009; Höner et al., 2023). A *broad* approach takes into account multidimensional sports performance data; physical, motor, and psychological characteristics; and sporting, professional, and family environments (Williams et al., 2020). A *dynamic* approach focuses on developmental trajectories and thus, necessarily uses multiple measurement points for adequately determining developmental potential (Güllich, 2020; Höner et al., 2023).

Choosing a broad approach recognizes that, in addition to motor characteristics, certain psychological characteristics and specific environmental conditions must be present to enable individuals to realize their potential. Conversely, a dynamic approach recognizes that relying on a single sport-specific performance measurement provides an insufficient understanding of individualized developmental trajectories and inadequate predictions of adult performance potential. Although the broad and dynamic approach for talent identification is undisputed today, it raises a number of conceptual questions:

1. As the broad approach assumes, if many criteria characterize talent, it is necessary to clarify how to handle such a “shopping list of criteria” (Williams & Reilly, 2000, p. 658): Which criteria are relevant? What interactions exist between different talent criteria? To what extent is compensation possible? How should we assemble criteria into an overall picture?
2. The dynamic approach considers developmental trajectories in specific talent dimensions (e.g., achievement motivation, familial support, competitive performance) as essential criteria for evaluating talent. However, does the relevance of the criteria change over time? How can we grasp the individuality of developmental trajectories across numerous measurement points?

Currently, there are few satisfactory answers to these questions (Höner et al., 2023), prompting us to question how well the nature of the phenomenon has been considered with current research strategies. In the broader context of psychological research, when confronted with unsatisfactory answers, Magnusson (1992) recommended the strategy of going “back to the phenomenon”, a strategy we will adopt in this paper. He summarized it as follows:

Today, I will argue for the supremacy of phenomena […]. My simple but forceful point is that the appropriate use of theory, method, and statistics in psychological research must be based on, and refer to, careful, systematic analysis and description of the phenomenon *per se*. If this rule is not maintained, we will go on producing data but the contribution to our understanding of why individuals [develop] as they do in real life will be much less than it could or should be. (Magnusson, 1992, p. 2)

### Description of the phenomenon and problem

Talent identification in sport examines a *developmental phenomenon*. Specifically, it aims to understand how each *individual develops* between (at least) two measurement points: t_1_ (as a child) to t_2_ (as an adult) (Conzelmann et al., 2018). Therefore, it seems logical to (a) situate talent identification within *developmental science*, defined as the inter-disciplinary science of all human-related phenomena that develop over time (Dick & Müller, 2017a), and (b) apply developmental-theoretical perspectives to the *problem* of talent identification research.

**Figure 1:**
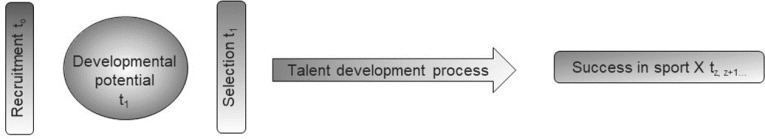
Basic talent research problem (Conzelmann et al., 2018, p. 88), reprinted and adapted with permission from the publisher Springer Fach-medien Wiesbaden GmbH.

The *problem* of talent identification research lies in assessing individuals’ potential for international success in adulthood (see Figure 1). Fundamentally, this problem is *predictive* in nature, involving a differential developmental *prediction*—discerning who will evolve into a professional and who won’t. Consequently, returning to the phenomenon and recognizing its developmental nature will provide a useful framework for shaping specific research *goals* and *conceptual contexts*.

### Developmental-theoretical perspective

#### Research goals

Developmental science works toward four central goals “to describe, explain, predict, and optimize changes in individuals across the life span” (Lerner & Bornstein, 2021, p. 1). Comparatively, *talent identification* in sport aims to describe, explain, and predict those advancing to higher performance levels. However, compared to general human development which examines how humans develop in general, talent identification research focuses on differential human development to identify who develops in specific ways (Zuber et al., 2016), and more particularly, into outstanding positive outliers.

#### Conceptual context

Over the last 50 years, developmental science unraveled a conceptual context with strong (meta-)theoretical foundations (Overton, 2015b; Reese & Overton, 1970), which are now widely undisputed (Lerner, 2021b). Within this conceptual context, “all data are theory-laden” (Dick & Müller, 2017b, p. 4). As such, results derived from the central goals of developmental science of describing, explaining, predicting, and optimizing human development are always part of a prescriptive or “nested hierarchy” (Witherington et al., 2018, p. 183). Reese and Overton (1970) explain nested hierarchy as follows:

Any theory presupposes a more general model according to which [...] theoretical concepts are formulated. At the more general levels, the concepts are generally less explicitly formulated, but they nonetheless necessarily determine the concepts at lower levels. This categorical determinism stretches from metaphysical and epistemological levels "downward" through scientific theories, to the manner in which we analyze, interpret, and make inferences from empirical evidence. (Reese & Overton, 1970, p. 117)

**Figure 2:**
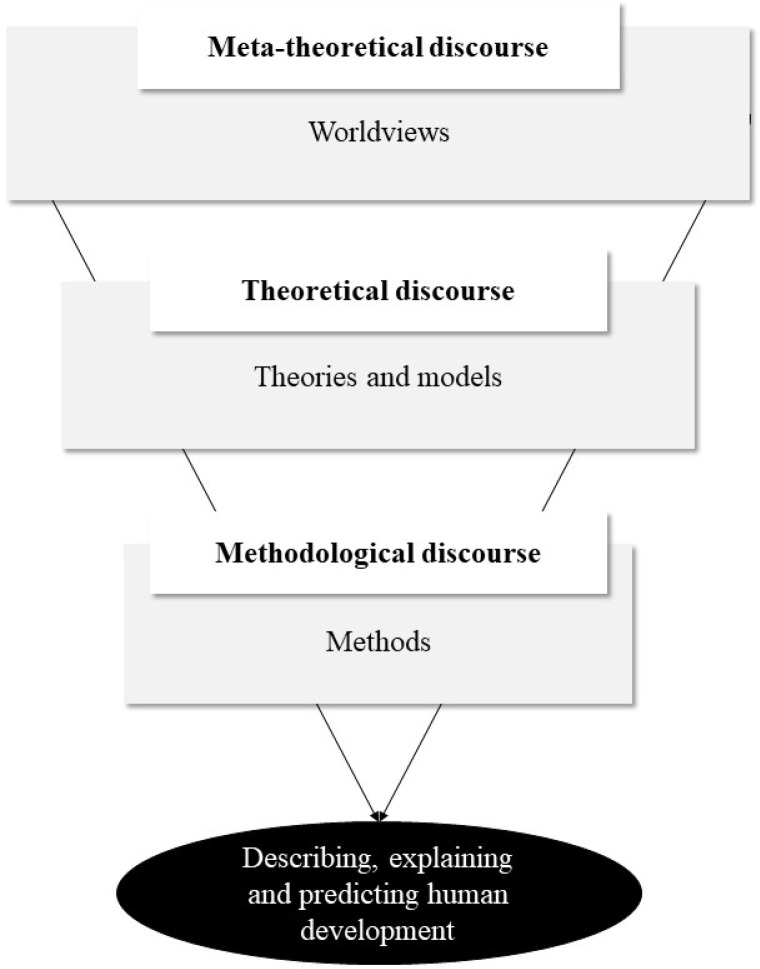
*Conceptual context from developmental-theoretical perspective: the nested hierarchy. Adapted from Figure 1 from Overton, W. F. (2014). Commentary: The process-relational paradigm and relational-developmental-systems metamodel as context.* Research in Human Development, 11*(4), 323–331. Copyright © Taylor & Francis Group, LLC (2014), reprinted by permission of W. F. Overton and Informa UK Limited, trading as Taylor & Francis Group. Available at:* http://www.tandfonline.com.

At the highest place in the nested hierarchy is the meta-theoretical level of worldviews (see Figure 2). A *worldview* “presents a vision of the nature of the world and the nature of how we know that world” (Overton, 2013, p. 26). Accordingly, a worldview constrains the type of *theorization* shaping the object of inquiry at the theoretical level; prescribes specific *methods* for the developmental analysis at the methodological level and conditions the type of *question* asked (see for example, Lerner, 2007; Overton, 1984, 2007).

Two worldviews exist in developmental science: the Cartesian-split-mechanistic worldview and the processual-relational worldview (Overton, 2013). Surprisingly, to our knowledge, there is no explicit reference to these world-views within the field of talent identification research in sport. In particular, the critical evaluation of their *fidelity to the phenomenon* of talent, a practice noted as the first requirement of science (Freeman, 2007; Magnusson, 1992), has never been undertaken. In the following sections, we portray metatheoretical assumptions, theoretical and methodological consequences, and main research directions of each worldview. Then, we describe implicit applications of each worldview in talent research and discuss the benefits and limitations of such applications with regards to their fidelity to the phenomenon (Freeman, 2007; Magnusson, 1992).

### The Cartesian-split-mechanistic worldview

#### (Meta-)theoretical assumptions

The Cartesian-split-mechanistic worldview is guided by three core principles: (1) decomposition; (2) foundationalism and atomism; (3) additive linear causal recomposition (Overton, 2013). Inspired by René Descartes (1596-1650) who *split* the body from the mind and created a long tradition of decomposition as dichotomization (Kowalski & Mrdjenovich, 2016), the principle of *decomposition* is an intellectual strategy to dissociate components of a whole. Yet “in order to split, one must accept the twin principles of foundationalism and atomism” (Overton, 2007, p. 31). *Atomism* presupposes all complex phenomenon “can be reduced to foundational discrete elements” (Overton, 2014, p. 22), in which discrete means “independent” from each other (Witherington & Heying, 2013, p. 164). However, the atomization process is not endless, as for reductionists, *foundationalism* allows for a “final fixed secure base” in the end (Overton, 2013, p. 38)—the atoms constituting the phenomenon. Finally, *additive linear causal recomposition* postulates decomposed and split parts can be entirely recomposed and whereby “all complexity is *simple complexity* in the sense that any whole is taken to be a purely additive [and linear] combination of its elements” (Overton, 2007, p. 31).

Interestingly, the procedures (e.g., decomposing, isolating, and manipulating the basic elements) mirror how social-behavioral research, psychological research, and, as we will demonstrate, talent research, primarily adhere to the *natural sciences* model (Salvatore & Valsiner, 2010) and its associated *hypothetico-deductive*, top-down procedure (Martini, 2017). To our knowledge, such adherence to the natural science model traces back to Windelband (1904), who labelled psychology as “*geistige Naturwissenschaft*” or “the natural science of the mental” (p. 11). He observed that “its entire procedure, its methodological arsenal, is from beginning to end that of the natural sciences” (p. 11).

Finally, as the third adjective suggests, the Cartesian-splitmechanistic worldview conceptualizes the nature of the world in a *mechanistic* manner, viewing it as a (dis)assemble-able aggregate and employing a *machine* as a guiding metaphor (e.g., clockwork) (Pepper, 1942). This results in a machine-like “model of [hu]man” (Reese & Overton, 1970, p. 131) where humans are conceptualized as “reactive, passive, robot” (Reese & Overton, 1970, p. 131). As we describe in the next paragraphs, such conceptualizations possess theoretical and methodological consequences, which steer research in the direction of a quest for nomothetic statements.

### Theoretical consequences

#### Variable-oriented theorization

Theorizations in the Cartesian-split-mechanistic world-view favor a monodisciplinary approach by reducing development to specific variables (pieces of the machine). For each piece, there is a dedicated specialist mechanic who meticulously examines it under a microscope, endeavoring to comprehend its relevance and establish its relationships with other pieces for example, a geneticist for the genetic pieces (Breitbach et al., 2014; Polderman et al., 2015); a psychologist for the psychological pieces (Ivarsson et al., 2020; Wachsmuth et al., 2023); a physiologist for the physiological pieces (Dodd & Newans, 2018; Murr et al., 2018). Consequently, theory construction begins by focusing and mapping categories of behavior, variables, or functions, resulting in function-centered or variable-oriented theorizations (Baltes et al., 2007; Vondracek & Porfeli, 2002).

#### Homogeneity assumption

Variable-oriented theorizations are built upon the premise of the “homogeneity assumption” (Richters, 2021, p. 368), which posits that all people share the same mechanical properties with respect to how variables are associated with each other. Like “uniform and fixed” *robots* (Overton, 2007, p. 30), individuals are assumed to be isomorphic or “interchangeable members of a single class” (Richters, 2021, p. 372); individuals are nothing more than “replication devoid of individuality” (Molenaar & Ram, 2009, p. 256). Accordingly, they function and develop through the same program or developmental model; each piece of the machine or each variable is thought to have the same fixed and invariant effect for all individuals (Laursen & Hoff, 2006).

### Methodological consequences: Population- and variable-oriented methodologies

On the methodological level, the traditional proclivities for the natural sciences model and homogeneity assumption result in centering research around populations and variables, not individuals. Indeed, if humans are seen as homogenous, it seems logical to mostly consider individual differences as just noise (Salvatore & Valsiner, 2011).

In variable-oriented *quantitative* methods, interest lies in population differences based on mean differences, or knowing populations through (curvi-)linear variable associations, such as variable A influences variable B (Overton & Lerner, 2014; Richters, 2021). Accordingly, population- respective variable-oriented methodologies (see General Linear Model [GLM]; Field, 2018), such as analysis of variance (ANOVA), multivariable analysis of covariance, correlation, or regression analysis, are favored. The GLM methodologies are commonly found in textbooks (e.g., Field, 2018) and systematically taught in academic settings (Bortz & Schuster, 2010). Similarly, when employing variable-oriented *qualitative* methods, the interest lies in identifying themes or variables that are common across all individuals (Gabler & Ruoff, 1979), as seen in approaches like thematic analysis (Clarke et al., 2015).

### Research direction: Quest for nomothetic statement

Ultimately, variable-oriented theorizations, homogeneity assumptions, and population- and variable-oriented methodologies lead to quests for one-size-fits-all *nomothetic* statements true for all people (Lerner & Lerner, 2019; Salvatore & Valsiner, 2011; Windelband, 1904). Nomothetic statements seek to reconstruct permanent, general, and invariable reality (Schläfer, 1999). As we will demonstrate in the upcoming section, talent identification research is replete with statements of such nature.

### Applications in talent research

**Figure 3:**
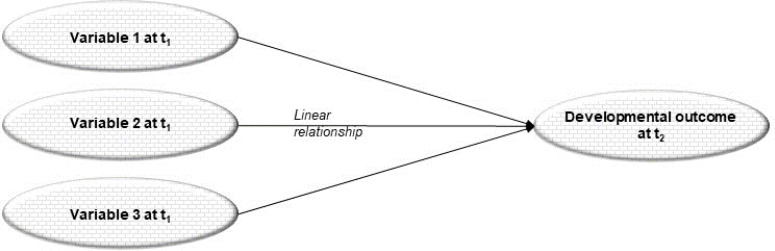
Talent reconstructed from Cartesian-split-mechanistic worldview (Conzelmann et al., 2018, p. 95); reprinted and adapted with permission from the publisher Springer Fachmedien Wiesbaden GmbH.

What applications, benefits, and limitations emerge from the Cartesian-split-mechanistic worldview for talent identification research in sport? Although links between Cartesian-split-mechanistic worldview and talent identification research have not been explicitly made to-date, the talent phenomenon is very often seen as a machine-like, (dis)assemble-able aggregate we can reduce to its smallest pieces (i.e., atomization in talent predictors), and recompose as the sum of weighted independent parts (Figure 3; see also Höner et al., 2021; Sieghartsleitner et al., 2019).

From a theoretical standpoint, prominent talent models such as in Gagné (2005), Heller et al. (2005), Williams et al. (2020) or Höner et al. (2023) can be characterized as variable-oriented theorizations. These models are typical re-finements of Figure 3, which focus on and map relevant tal-ent categories and variables. In terms of methodology, closer examinations of the empirical discourse revealed that the *General Linear Model* constitutes the predominant approach employed in the majority of research studies (for reviews, see Murr, Feichtinger, et al., 2018; Owen et al., 2022). Thus, to date, the Cartesian-split-mechanistic worldview has been the most popular *modus operandi* of talent research. As we will outline below, the Cartesian-split-mechanistic research agenda in talent research revolves primarily around the *search for talent criteria*. This search involves four variable-oriented research questions aiming toward nomothetic statements:

1. What *variables* distinguish between different developmental outcomes?
2. Which *variable* is more important?
3. What *variables* possibly confound predictions?
4. Can we correct the confounding influences of these *variables*?

It is unrealistic to review the whole of talent identification research regarding these four questions. Our examples illustrate, we do not claim completeness.

#### What variables distinguish between different developmental outcomes?

In talent identification, the developmental outcome of interest is the career outcome, such as expert versus non-expert (Bergkamp et al., 2019) and researchers search for key pieces of the talent machine with predictive validity (Gabler & Ruoff, 1979; Johnston & Baker, 2022). Over 20 years ago, Williams and Reilly (2000) provided a theoretical list of key predictors, i.e., the mechanical talent properties shared by all people. Since then, several reviews underlined the empirical usefulness of physical (Rees et al., 2016), physiological (Dodd & Newans, 2018), psychological (Ivarsson et al., 2020; Wachsmuth et al., 2023), sociological (Hauser et al., 2022; Reeves et al., 2018), training-related (Baker & Young, 2014; Charbonnet & Conzelmann, 2023), and technical (Murr, Feichtinger, et al., 2018) variables for developmental predictions. All distinguish between two populations of athletes with quantitative variable differences, the population of future professionals possess better *mean* values than the pop-ulation of future non-professionals.

#### Which variable is more important?

After disassembling the “talent machine”, researchers tend to investigate which variable is ‘bigger’, thereby seeking to find “the most important contributors” (Kite et al., 2021, p. 1) or “the most important indicators” (Rogers et al., 2022, p. 1175) for performance and development. For instance, reseachers have debated between the importance of nature or nurture (e.g., Issurin, 2017; Zempo et al., 2019); technical skills or functional capacities (e.g., Sieghartsleitner et al., 2019); personality traits or environmental characteristics (e.g., Fuhre et al., 2022; Larkin & O’Connor, 2017). Ultimately, comparing variables of importance lead to the search for “appropriate algorithms and weightings” for a talent identification model (Abbott & Collins, 2002, p. 161). *Weighting* can be derived from different sources of information with multiple regression analyses (e.g., Hohmann & Siener, 2021; Höner et al., 2021; Sieghartsleitner et al., 2019), meta-analytic estimates (Neumann et al., 2023), or expert judgement (Hohmann & Siener, 2021; Höner & Votteler, 2016; Siener et al., 2021). For instance, Höner et al. (2015) summarized and formulated technomotor performance of football players: “score = 10,000 * [(17.29 * sprint) + (9.43 * agility) + (4.11 * dribbling) + (2.41 * ball control) + shooting]-1” (p. 3). Similar strategies are found in Switzerland where the nationwide talent identification model is based on adding weighted variables derived from expert judgements to a total score (Fuchslocher et al., 2016). In such talent identification models, inputs are strictly proportional to output wherein each variable has the exact same weight for all individuals in predicting who might develop into professionals: Talent is described with a function. This results in a one-size-fits-all recipe and everything revolves around the pragmatic search for nomothetic regularities: the same talent model for all.

#### What variables possibly confound predictions?

A separate group of studies found problems with the talent machine, i.e., variables that confounded predictions of athlete future chances of success (Jung, 2022; Wattie et al., 2014). The most examined confounding variables are probably relative age (Leyhr et al., 2021; Romann et al., 2018); maturity status (Cripps et al., 2016; Figueiredo et al., 2009; Malina & Cumming, 2004); and training age (Guimarães et al., 2019; Valente dos Santos et al., 2014). All confounding variables provide task-dependent advantages for one population of athletes compared with others. For example, in sports like soccer, alpine skiing or tennis, those who are relatively older, are earlier mature, or those with more training experience tend to be advantaged and this generates selection errors in talent selection systems (Johnston & Baker, 2020; Romann et al., 2018).

#### Can we correct the confounding influences of these variables?

To counter such selection errors, some researchers strictly followed the implicit logic of the machine metaphor suggesting that if all athletes are like homogenous machines, we can identify and/or quantify bias present in the machine and, we can highlight and/or remove problematic pieces and correct the bias.

*Identifying and highlighting*. Some researchers proposed strategies such as highlighting or grouping the problematic piece of the machine for coaches by player-labelling (Lüdin et al., 2022; Mann & van Ginneken, 2017) or biobanding (Cumming et al., 2017; Malina et al., 2019). While the former makes confounding variables such as maturity status explicitly visible by athletes’ shirt numbers, the latter groups athletes according to advanced, on time, or delayed maturity status to better observe performance.

*Quantifying and removing*. Other researchers developed correction mechanisms to deal with the confounding influences of problematic variables. In a first step, correction mechanisms quantify the influence of confounding variables on performance tests. Then, they adjust player performance scores to mirror player expected performance without developmental (dis-)advantage (e.g., Charbonnet et al., 2022; Larochelambert et al., 2022; Romann & Cobley, 2015). These correction mechanisms promise unbiased interindividual performance comparisons thus, unbiased summaries of weighted variables.

### Benefits

By focusing on variable-oriented and (global) population-level summaries while striving to generate nomothetic knowledge, Cartesian-split-mechanistic analysis addresses several of the conceptual questions raised in the introduction regarding the broad and dynamic approach to talent identification. Three main benefits of the Cartesian-split-mechanistic analysis stand out: (1) search for talent criteria, (2) comparative insights regarding the relevance of talent criteria, and (3) empirical success compared to human intuition.

1. The analysis serves as valuable starting point for the scientific journey to examine the talent phenomenon. Indeed, it is only possible to identify talent if we previously identified relevant talent variables, thereby giving substance to the broad approach (Sarmento et al., 2018; Williams et al., 2020).
2. The analysis estimates and compares the weight (isolated predictive effect) of each talent criterion for the average person within the sample, shedding light on whether and how the significance of talent criteria evolves over time (Baker et al., 2019).
3. The analysis assembles criteria additively into an overall picture (aggregated model; Meijer et al., 2019). Based on general decision-making literature (see Grove et al., 2000; Kahneman et al., 2021; Meehl, 1954), this type of assembly provides insight into *additive* compensation possibilities and enhances decision-making when compared to human intuition. In the specific area of talent research, the handful of studies that exist align with this overarching trend (e.g., see Sieghartsleitner et al., 2019).

### Limitations

Fifty years ago, Carlson (1971) asked, in the context of psychology in general, “where is the person in personality research”? (p. 203). In doing so, she criticized the unexamined orientation toward what we refer to as the Cartesian-split-mechanistic worldview (e.g., natural sciences model, focus on *variables*), which led to an inadequate consideration of the *person* as the phenomenon. The research community had simply applied the methods they were traditionally socialized with, without knowing that the machine model and its associated methodologies were *mismatched* with the properties of phenomenon (Magnusson, 1992). This could be a result of secondary ignorance (Eisner, 2005, p. 139), signifying not knowing something, but you do not know that you do not know (as opposed to primary ignorance when you do not know something, but you know that you do not know it).

This mismatch critique also applies to the specific context of talent identification research whose methodological approach has not been dictated by the nature of the talent phenomenon. Rather, the widespread use of the GLM methodology has molded our understanding of the phenomenon, introducing notable limitations: (1) the specificity problem and (2) the complexity problem.

#### Specificity problem: Infidelity to primary focus of talent research (nomothetic versus idiographic; aggregate versus individual)

As noted in the introduction, talent identification focuses on *differential* human development, seeking to understand what makes us *heterogenous*, unique and different from one another rather than on general human development (seeking what makes us *homogenous*). Such focus underscores the imperative to furnish practitioners with idiographic-, person-oriented answers, referable to *individuals*.

Paradoxically, despite this emphasis on the *person*, talent identification research leans towards the Cartesian-split-mechanistic worldview, which starts by definition with the assumption of homogeneity (Richters, 2021), leading to *variable*-oriented methodology, and consequently constructing *nomothetic*-oriented prediction models. However, the only “person” revealed by such models is “an abstract and entirely fictional entity whom the Belgian polymath Adolphe Quetelet (1796–1874) famously labeled *l’homme moyen*, or the average man” (Lamiell, 2019, p. 282). This entity is essentially an *aggregate*, a statistical construction: the “average talent.” Consequently, prediction models crafted in the Cartesian-split-mechanistic worldview (e.g., multiple linear regression) assume (1) that talent criteria possess the same meaning for all (i.e., weight or predictive effect found for the average talent) and (2) that findings about the aggregated “average talent” (population level) are suitable to generate individual selection recommendation (individual level). But do these assumptions hold true?

Contemporary theoretical reflections suggest the contrary, underscoring a *specificity* problem (Bornstein, 2017): Talent variables might possess different meanings for different people in different contexts. In fact, as Richters (2021) stated, “in a world populated by heterogeneous individuals—the world we live in—variables do not and cannot have fixed, invariant, inherent causal properties” (p. 387). Thus, there are no guarantees that prediction models for an aggregate (population level) hold true for individual cases (individual level) (Bergman & Wångby, 2014). For example, for a specific person, the talent criterion A might be far more important than talent criterion B, whereas the prediction model for the aggregate assumes otherwise. Although researchers caution against universally applying population-level trends to individuals (Molenaar, 2004; Salvatore & Valsiner, 2010), sport federations and practitioners are often attracted to the pragmatic appeal of an aggregated, one-size-fits-all prediction model (see, for example, Fuchslocher et al., 2016).

**Figure 4:**
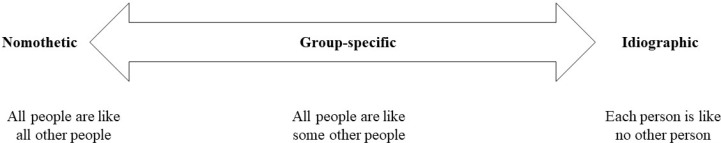
The three kinds of laws of human development.

At its core, talent identification is about evaluating and selecting one (specific) person, not a (general) aggregate. Thus, to adequately serve its objective of exploring individual differences, talent identification research should shift the focus from a quest for nomothetic knowledge to a quest for idiographic knowledge, and consequently, from prediction models for the aggregate to prediction models for the person. This resonates with Kluckhohn and Murray’s claim (1948), made over 70 years ago: Because “all people are like all other people, all people are like some other people, and each person is like no other person” (as cited in Lerner, 2007, p. 7), we require three kinds of laws of increasing specificity to fully understand human development: nomothetic, group-specific, idiographic (Runyan, 1983; Windelband, 1904) (Figure 4).

#### Complexity problem: Infidelity to nature of “whole” (mechanical versus dialectical)

Fuchs-Kittowski (1981) distinguished between two kinds of whole: *mechanical* (whole as the mere sum of its constituent parts) or *dialectical* (whole is more than the sum of its parts). In a mechanical whole, complexity is simple, linear, and additive, such as 1 + 1 = 2. In contrast, in a dialectical whole, complexity is complex, emergent, and autopoietic, such as 1 + 1 = yellow (Balagué & Torrents, 2005). By definition, Cartesian-split-mechanistic talent identification constructs a *mechanical* whole—namely, a prediction model where talent criteria are weighted and summed into a total score (e.g., Sieghartsleitner et al., 2019). Such a prediction model aligns with the machine metaphor and utilizes the *additive linear causal recomposition principle* to reconstruct the talent phenomenon. However, the nature of the talent phenomenon might not be compatible with the mechanical whole, introducing a *complexity* problem.

There are situations where the summation provides a complete understanding of the phenomenon; for instance, the *mass* of a whole being the sum of its individual parts (Medawar & Medawar, 1983). However, following the movement of thought from Gestalt theory (Koffka, 1936) to ecological, biological, and physical sciences (Hickel, 2020), there are also numerous situations where the whole may actually be *more* than the sum of its parts. In particular, when aiming to predict the development of an individual, Magnusson and Törestad (1993) stated that “whole picture has information value that is beyond what is contained in its specific parts” (p. 436). Hence, forecasting which individuals will reach professional status in sports using a mechanical whole may not accurately capture the true nature of the talent phenomenon.

**Figure 5:**
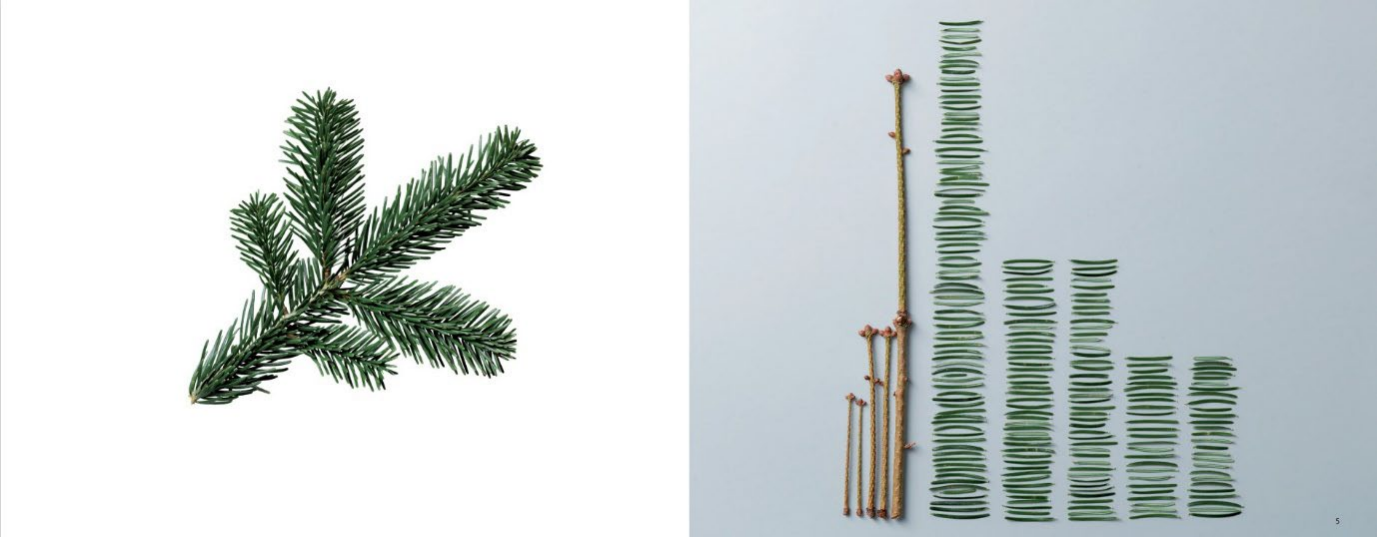
**Kunst aufräumen** by Urs Wehrli, Copyright ©2011 by KEIN & ABER AG Zürich – Berlin. The left panel shows the whole as more than the sum of its parts (holism, complex complexity, person-oriented focus); the right panel shows the whole as the sum of its parts (reductionism, simple complexity, variable-oriented focus).

The artwork of Urs Wehrli vividly illustrates this complexity problem. In *Kunst aufräumen* (tidying up art; Figure 5), a fir branch is presented in the left panel as a metaphor for the talent phenomenon. The right panel showcases its decomposition into parts. The decomposition process—from the left to the right side—is easy; we can atomize talent to identify the single parts in the mechanistic, variable-oriented and nomothetic approach, then weight these parts, and add them through additive recomposition (*mechanical* whole, e.g., multiple linear regression). However, such additive recomposition does not maintain the organismic beauty of the holistic Gestalt of the left side (*dialectical* whole)—the unique art or design gets lost in the additive recomposition. Therefore, to avoid losing information and capture the true nature of the talent phenomenon, talent identification research needs a shift from mechanical to dialectical whole, from analytical recomposition approaches to more holistic, global ones; for example, system-oriented approaches considering emergent, self-organized, dynamic interconnectedness parts (Balagué & Torrents, 2005; Balagué et al., 2017).

### The processual-relational worldview

#### (Meta-)theoretical assumptions

To capture the shifts outlined in the previous section, developmental science proposed leaving the Cartesian paradigm for another system of thought: the processual-relational worldview (Overton, 2013). The *processual-relational worldview* is guided by core principles diametrically opposed to the Cartesian-split-mechanistic ones: (1) non-decomposability (instead of decomposition); (2) holism (instead of atomism and foundationalism); and (3) emergence and nonlinear recomposition (instead of additive linear causal recomposition).

The *non-decomposability principle* “finds its historical origins in Aristotle’s insistence that form and matter cannot be separated into two discrete elements” (Overton, 2013, p. 42) and its origin in psychology in the idea of “indivisible” whole (Stern, 1911, p. 19). Non-decomposability rejects the dichotomous Cartesian question of *what is more important* by positing a dialectical model of reality where everything exists through continuous bi-directional exchanges (Keller, 2010; Moore & Shenk, 2017)—“a ceaseless flux” of interrelated changes (Lerner & Busch-Rossnagel, 1983, p. 3).

The *holism principle* encourages distance from the study of the variables of a whole in isolation and suggests rather starting theory construction “with consideration of the person as a system” (Vondracek & Porfeli, 2002, p. 387). In this system, the variables and the wholes are thought to stand in holistic relation to each other. Figure 5 precisely shows the holistic relations that make the painting a *work of art*. Overton and Lerner (2014) offer more clarity:

Parts get their meanings from wholes, wholes get their meanings from parts, and wholes differ in novel ways from the sum of their parts. Wholes and parts interpenetrate, interdefine, and fuse, and it is thus meaningless to consider constructing or deconstructing the whole by adding or subtracting parts. Rather than isolated pure forms interacting, organic wholes coact and coconstruct. (Overton & Lerner, 2014, p. 68)

Accordingly, the holism principle replaces the *machine metaphor*—and its focus on the sum of single variables—with the metaphor of an active, living organism, such as a plant (Pepper, 1942, 1943), and a focus on an *organized totality*, a Gestalt (Figure 5, left panel): a system made of ongoing interaction processes on multiple levels (Reese & Overton, 1970). Rather than building a *mechanical* whole, the holism principle opts for creating a *dialectical* whole (Fuchs-Kittowski, 1981).

Finally, the *emergence and nonlinear recomposition principle* is a logical consequence of a complex world governed by holism. If the whole is more than the sum of its parts, discontinuity and nonlinearity should be expected (“transformational changes”; Overton, 2015a, p. 19). New and unexpected states may therefore emerge from the dynamic inter-action of the parts (Dai, 2005; Greenberg et al., 2013).

Altogether, the processual-relational worldview results in a “model of [hu]man” (Reese & Overton, 1970, p. 131) where organisms are “inherently active, self-creating, self-organizing, self-regulating (agentic), nonlinear and complex, and adaptive” (Lerner & Lerner, 2019, p. 63).

### Theoretical consequences

#### System-oriented theorizations

The processual-relational worldview shifts the unit of analysis from aggregates to a *complex dynamic system* (Lundh, 2015)—like precursors such as Allport (1961) proposed more than 60 years ago. Complex dynamic systems are interdependent networks of forces characterized by two principles: The relational principle, which posits constant interconnectedness (Magnusson, 1985), and the adaptive principle, which assumes constant changes (Hiver et al., 2021; Magnusson, 2014). As such, a complex dynamic system is inherently never at rest and produces knowledge about human development that always applies to specific person-context-assemblies across time or *indivisible totality* (Magnusson & Stattin, 2007). The goal is to describe “how [one] state develops into another state over the course of time” (van Geert, 2009, p. 244). Accordingly, researchers usually ask “change-oriented, relational questions, questions that bridge levels of analysis and that require multidisciplinary collaboration for their answers” (Lerner, 2021a, p. 107).

#### Specificity principle

A regulative tenet of the system-oriented view is the specificity principle. The *specificity principle* posits that “specific outcomes in human development always involve coaction of specific individuals at specific times in specific places through specific processes” (Lerner & Bornstein, 2021, p. 1). The specificity principle contrasts with the Cartesian homogeneity assumption and the idea that human development is explainable by the same model for all individuals. Instead, it assumes that specific experiences affect the development of specific people in specific contexts at specific times in specific ways. Therefore, no entity or variable, has fixed, invariant and context-free properties which can be identified in all individuals (Bergman et al., 2003). Each entity always derives its meaning from the embedded context. In short, developmental research is about investigating which system state—what person in which context at a particular developmental stage—is likely attracted to which system state next (Guastello et al., 2009).

### Methodological consequences: System-oriented methodologies

System-oriented views typically employ either *network*- or *person*-oriented methodologies to describe, explain, and predict system state from one measurement point to the next (Hiver et al., 2021).

#### Network-oriented methodologies

*Network-oriented* approaches, such as deep neural network (DNN; Goodfellow et al., 2016; Nielsen, 2015; in talent research, Pfeiffer & Hohmann, 2012) or dynamic network modelling (Strogatz, 2001; in talent research, Den Hartigh et al., 2016), generate developmental predictions based on an *organismic* model (LeCun, 2019). Just like the human nervous system, network-oriented approaches consist of connections between nodes. Furthermore, they usually use several iterations of trial and error to learn, and are able to consider *nonlinear* interactions (LeCun et al., 2015). In short, network-oriented approaches focus on the developmental system and its “complex underlying structure of interdependent relations” (Hiver et al., 2021, p. 4).

#### Person-oriented methodologies

On the methodological level, the specificity principle requires centering research around individuals (difference between types of persons), not populations or variables (Bergman & Andersson, 2010; Bergman & Lundh, 2015; Lundh, 2019). *Person-oriented* approaches use qualitative methods such as structural narrative analysis (B. Smith & Sparkes, 2009 ; in talent research, John & Thiel, 2022a) and quantitative methods such as the LICUR method (Bergman et al., 2003; in talent research, Zibung & Conzelmann, 2013) to disaggregate the population into several *types* of persons, which function and develop according to different laws; thus, they follow different developmental paths (Bergman et al., 2003; Bergman & Wångby, 2014; von Eye et al., 2015). In contrast to the network-oriented methodology, the person-oriented methodology illustrates individual “trajectories of change” (Hiver et al., 2021, p. 4), proposing different talent models for different individuals (Zuber et al., 2016).

### Research direction: A quest for idiographic statements

Ultimately, system-oriented theorizations, specificity principle, and network- and person-oriented methodologies lead to quests for *idiographic* statements true for specific person-context-assemblies (Lundh, 2022; Windelband, 1904). Idiographic quest represents a genuine effort to focus more on the particular, the contextual, and/or temporal rather than the general, context-free, and timeless (Luca Picione, 2015; Valsiner, 2016). As such, system-oriented views attempt to get to know the individuals and their specificity rather than general populations or isolated variables (Bogat et al., 2016; Lerner & Bornstein, 2021).

### Applications in talent research

For talent identification research, the question arises: What are the applications, benefits, and limitations of the processual-relational worldview for talent identification research?

Although the processual-relational worldview has often been claimed theoretically, it is rarely truly implemented on the methodological level in talent research (Balagué et al., 2017; John & Thiel, 2022b). Some examples of system-oriented *theorization* in talent research include bioecological models (Bronfenbrenner & Morris, 2007; in talent research Henriksen et al., 2010); holistic-interactionistic models (Magnusson & Stattin, 2007; in talent research Zuber et al., 2016); and system-/network-theoretic models of development (Guastello et al., 2009; in talent research, Den Hartigh et al., 2016; Pfeiffer & Hohmann, 2012). Furthermore, the processual-relational principles of non-decomposability, holism, and emergence/nonlinear recomposition are (implicitly) widely accepted in the scientific conceptualization of talent (Baker et al., 2019; Simonton, 1999; Zuber et al., 2016). In fact, it is hard to find empirical papers which do not mention one of these approaches either in introduction or discussion sections. However, processual-relational research *methodologies*, such as network-oriented and person-oriented approaches, are only observed in a minority of studies.

Rather, most proponents of the processual-relational theoretical approaches in talent identification research use GLM methodologies (e.g., multiple regression analysis, correlation, ANOVA) and overlook the crucial step of aligning their methods with the fundamental tenets of their theories (see for example the reviews of John & Thiel, 2022b; Murr, Feichtinger, et al., 2018; Owen et al., 2022). In other words, most empirical contributions in the field of talent research have a theory-method *mismatch*: They are often chimerical, meaning that they have processual-relational-oriented introduction, theoretical framework and discussion sections, yet a Cartesian-split-mechanistic-oriented methods and results section (using GLM). Considering this mismatch, our insights into talent identification research from a processualrelational worldview are somewhat limited. Nonetheless, there is a specific subset of studies with processual-relational methodologies, which are dedicated to addressing two fundamental questions:

1. To what extent does a network predict a developmental outcome?
2. What type of person is more likely to follow which particular developmental path?

#### To what extent does a network predict a developmental outcome?

Although artificial neural network methods are not the norm, they have been applied in talent identification research for over two decades (see Edelmann-Nusser et al., 2002; Rygula & Roczniok, 2004; Silva et al., 2007). These techniques have proven successful in predicting performance across various sports, including table tennis (Siener et al., 2019); tennis (Siener et al., 2021); soccer (Hohmann & Siener, 2018); swimming (over relatively short periods of 1 year; Maszczyk et al., 2012; and over longer periods of up to 5 years Allen et al., 2015) and predicting drop out of gymnastics (Pion et al., 2017). In general, predictions derived from artificial neural network methods surpass those made by traditional, linear methods (Pfeiffer & Hohmann, 2012; Pion et al., 2017). Consequently, network-oriented research expands our understanding by enhancing predictive capabilities.

In parallel, Dynamic Network Modelling has been employed as an approach to explain and predict the development of excellent human performance (Den Hartigh, Hill, & van Geert, 2018; Den Hartigh, Niessen, et al., 2018; Den Hartigh et al., 2016). In general, the simulations could “accurately predict the characteristic properties of excellence” (Den Hartigh et al., 2016, p. 3). For instance, a research group was able “to replicate the qualitative pattern of achievements of some exceptional athletes in different sports (i.e., Federer, Williams, Crosby, and Messi)” (Den Hartigh, Hill, & van Geert, 2018, p. 10).

#### What type of person is more likely to follow which particular developmental path?

**Figure 6:**
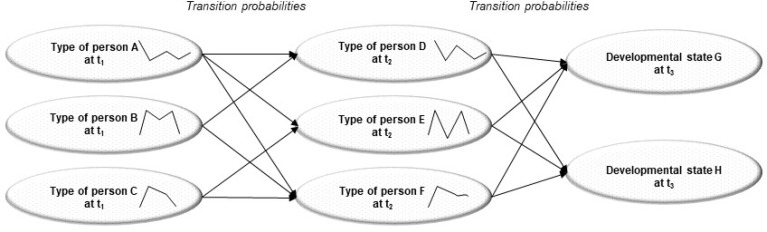
Talent reconstructed from processual-relational worldview with LICUR method (Conzelmann et al., 2018, p. 96); reprinted and adapted with permission from the publisher Springer Fachmedien Wiesbaden GmbH

To our knowledge, only one research group focused on trajectories of different types of individuals over time in talent identification research in sport science. They used the LICUR method to investigate idiographic-oriented trajectories at different developmental stages (age groups; Figure 6). Through clustering, they followed the directions different types of person are more likely to develop – where they are attracted (developmental type) or not (developmental antitype) – and whether or not new, unexpected states emerged at different time points. They provided results in team sports such as football (e.g., Zibung et al., 2016; Zuber et al., 2015, 2016) and ice hockey (e.g., Lenze et al., 2023; Stegmann et al., 2021), as well as individual sports (e.g., Schmid et al., 2021).

### Benefits

Three main benefits of the processual-relational type of analysis aid researchers in addressing several questions: (1) fidelity to the phenomenon, (2) identification of unprecedented compensation possibilities, and (3) empirical success compared to Cartesian-split-mechanistic worldview.

1. The widespread popularity of the processual-relational worldview on the theoretical level is unsurprising, as it genuinely aligns with the nature of the talent phenomenon: (a) this worldview conceptualizes individual as what they are—living organisms rather than machines; (b) it predicts development in the way the world works—non-linearly rather than linearly; and (c) it focuses on the relevant domain of inquiry—differential human development rather than general, population-oriented development. Simply put, the processual-relational worldview is most likely to do justice to the holistic assessment and the selection or de-selection of one individual.
2. In contrast to the Cartesian-split-mechanistic worldview, the processual-relational worldview goes beyond the simplistic examination of *additive* compensation possibilities. Network-oriented analysis explores compensation possibilities beyond human understanding, while person-oriented analysis reveals compensation possibilities beyond the sum of the parts. Indeed, certain individual configurations (see Figure 6), and “not necessarily those having the highest overall scores” (Zuber et al., 2016, p. 1), are more likely to succeed in the future compared to others (Conzelmann et al., 2018). Therefore, even if different individual configurations have the same total score in Cartesian-split-mechanistic analysis, a person-oriented approach can reveal different developmental paths and success probabilities for each type of person identified, emphasizing that the whole is more than the sum of its parts (Baker et al., 2018; John & Thiel, 2022a).
3. Processual-relational, nonlinear analysis integrates talent criteria into a dialectical whole. Based on general decision-making literature (see Kahneman et al., 2021), this type of assembly seems to enhance decision-making when compared to Cartesian-split-mechanistic, linear predictions. In the specific area of talent research, the handful of studies that exist seems to align with this overarching trend (e.g., see Pfeiffer & Hohmann, 2012; Pion et al., 2017).

### Limitations

Despite these considerable benefits, network- and person-oriented methodologies are not immune to criticism. As we will elucidate, network-oriented approaches have transparency and moral problems, while person-oriented approaches struggle to implement the holistic idea and remain static snapshots of the person-environment-system dynamic.

#### Transparency problem

Nonlinear machine learning algorithms stand in opposition to more traditional, transparent, additive rule-based algorithms (Gigerenzer, 2022): They “are black boxes, their workings are opaque, and their decisions cannot yet be explained or justified” (Beisbart & Räz, 2022, p. 1). We may know their parameters or variables, yet we do not understand the network dynamic (Gigerenzer, 2022). Therefore, the machine learning algorithm may tell us the probability of future chances of success, but it does not improve our understanding of the path to success itself. The lack of transparency involves three risks:

- *A decision without control*: We ignore any unwanted bias the machine learning algorithms possibly contain and reinforce (Jobin et al., 2019).
- *A science without theory*: We may distance ourselves from theory-guided science and Popperian quests for truth (Popper, 2005). The authority for knowing what to do and who to choose possibly shifts from human instinct to machine learning algorithms: Just pour in data and let the algorithm tell you what you need to know, which opens the door to radical empiricism and possibly renders theory (development) obsolete (Koenig, 2019).
- *A coach without knowledge*: It is suggested that humans suffer cognitively in the long run, when reducing their cognitive efforts and making their lives easier with technological innovations (Koenig, 2019). For example, the transition from map reading to GPS reduced our space orientation abilities (Koenig, 2019). Similarly, if coaches just follow what the algorithmic black box told them, they may gradually lose skills in tasks related to talent identification (Woods et al., 2021).

#### Moral problem

The transparency problem rapidly becomes a moral problem if the context, in which black box algorithms are used, is sensitive. Indeed, “people subjected to algorithmic decisions arguably have a moral right to understand how the decisions came about” (Beisbart & Räz, 2022, p. 2). Talent identification is a highly sensitive context because it decides future developmental opportunities of a child. Consequently, artificial neural networks face moral problems (Jobin et al., 2019).

#### Holism problem

Clustering algorithms, such as LICUR, are not magical either. They have no choice but to hurt the non-decomposability and holism principles: “They must be restricted to a relatively small number of variables, and therefore to less extensive models; i.e. four to six so-called operating factors” in order to deliver interpretable clusters (Sieghartsleitner et al., 2019, p. 2). Therefore, they only capture isolated parcels of disembodied wholes, where specific parts are favored over other ones, and mostly chosen based on a population-oriented and variable-oriented approaches (Bergman et al., 2003; in talent research, Conzelmann et al., 2018).

#### Static snapshots problem

Until now, person-oriented approaches cannot fully meet expectations related to dynamic aspects of talent. They may be able to reconstruct developmental paths through snapshot-like alignments of developmental states, yet what happens between static moments at various life stages remains unclear: The true development process of living organisms remains hidden in a black box.

## Discussion

In this paper, we reconstructed talent identification research in sport science based on foundational theories from developmental science. Accordingly, we traced back to what the talent field did theoretically and methodologically and framed it within two implicit paradigmatic systems of thought: Cartesian-split-mechanistic and processual-relational worldview. Each worldview proposes talent identification models with different characteristics (Balagué & Torrents, 2005). The characteristics include linear input-output machine versus nonlinear systems; cause-effect relations versus interaction and self-organization; mechanical versus dialectical whole; analytic, variable-oriented views versus global, network- and person-oriented views; nomothetic generality versus idiographic individuality; and reductionism versus holism. Overton (2014) sums up the differences best,

if your methodology assumes that the living organism is a linear input-output machine with strictly decomposable parts, then you will be prone to exclusively develop and use linear additive methods such as... ANOVA... and regression models. If, on the other hand, your methodology assumes that the living organism is a nonlinear self- organizing dynamic system, you will be prone to employ consistent methods, such as, for example, […] nonlinear dynamics systems models. (Overton, 2014, p. 19).

Drawing on Kuhn’s (1970) reflections about the structure of paradigm shift development and scientific revolutions, developmental talent identification research seems to be in its infancy or a “period in which no single paradigm […] is sufficiently well accepted to guarantee a concerted research effort and, as a consequence, research activity proceeds in a somewhat ‘piecemeal’ manner” (Abernethy & Sparrow, 1992, p. 18). If two paradigms coexist, three scenarios are possible: they are (1) incomparable and incompatible (*incommensurability*); (2) comparable (*competitivity*); or (3) compatible (*complementary*).

### Incommensurability

For those who agree with the first scenario, since comparing or combining the two worldviews is not possible, our paper ends here. We must accept that there are two irreconcilable ways of looking at the world (Bergman & Trost, 2006), which involve different criteria for truth, and thereby different theoretical and methodological understandings of what talent is (Reese & Overton, 1970). These two different understandings are incommensurable (Stegmüller, 1986)—incomparable and incompatible— and “there can be no fruitful debate between them” (Reese & Overton, 1970, p. 129).

### Competitivity

Others possibly believe some debate is fruitful. For instance, we can “compare the progressiveness of different research traditions [worldviews], even if those research traditions are utterly incommensurable in terms of the substantive claims they make about the world!” (Laudan, 1977, p. 146). Such comparison is not about arguing if one worldview is true or false; instead, it is about arguing which best suits the goals of talent research, such as predicting developmental outcomes (Laursen, 2015). As already mentioned, the research groups tackling such an issue in talent research found a nonlinear—processual-relational—method displayed better predictive results than linear-—Cartesian-split-mechanistic—methods (Pfeiffer & Hohmann, 2012; Siener et al., 2021).

### Complementarity

The last scenario focuses on complementary relationships between the two worldviews. For instance, von Eye and Spiel (2010) claimed two worldview agendas “do not differ to the extent that they cannot be related to each other” (p. 152). Since “no paradigm is perfect, and none is capable of identifying let alone successfully answering all the questions relevant to a given domain of inquiry” (Richters, 2021, p. 392), both likely complement each other. Such idea stands out empirically, with Siener et al. (2021) concluding that the most effective predictions come from combining a processual-relational, non-linear method with a Cartesian-split-mechanistic linear one, and also enjoys broad acceptance within the research community. For instance, person-oriented researchers such as Bergman et al. (2003) emphasized “from the beginning that the variable approach and the person approach are complementary, not contradictory” (p. 19). Similarly, Höner et al. (2023) wrote that “the person-oriented approach offers a conceptually convincing supplement to the established variable-oriented approach” (p. 553). In some cases, such complementarity may be more than something ‘nice to have,’ but a true necessity. As Windelband (1904) formulated more than a century ago, “idiographic [analyses] require, at every step, general theses, which they can borrow in their fully correctly established form only from the nomothetic” (p. 19). In that sense, person-oriented talent researchers borrow talent criteria identified with variable-oriented approaches for their analyses (Zuber et al., 2016).

## Toward a complementary research agenda

If we embrace the complementarity view, we should reflect on when we need one worldview and when we need the other when navigating through the maze of talent research. Interestingly, we noticed each worldview follows different roads for its analyses of talent phenomenon, yet cross halfway. As we explain next, both worldviews should not go farther than crossing in the middle, recognizing their limitations, and letting the other serve as a complement.

### Mapping the road 1: Top-down to group-specificity, but not farther

The Cartesian-split-mechanistic worldview starts the developmental analysis from the top-down—from the general to the particular—by keeping a nomothetic focus as long as possible. To understand the talent phenomenon, this worldview deconstructs the general structure of talent into particular pieces with predictive validity, searches for problematic pieces (Cumming et al., 2012), and applies the same general laws, such as weighting, to every particular individual (e.g., Sieghartsleitner et al., 2019).

Nevertheless, at some point in the analysis, researchers typically recognize the limited scope of a truly nomothetic focus and underline the need to refine its level of granularity. For instance, Höner et al. (2023) wrote that there is no doubt “the prognostic relevance of talent predictors […] for future success cannot be universally quantified for all development phases, sports, and performance levels” (p. 552). Such statements represent a move from nomothetic to group-specific level. Group-specificity is achieved by splitting the whole group or population with regard to one or more theoretically meaningful moderator variables, such as sport, age, performance level, personality, to obtain subgroups (Charbonnet & Conzelmann, 2023). The intention is to make more differentiated statements while staying close to the nomothetic pole. Therefore, the goal is the same prediction model for a group of people. Please note the nomothetic idea of plurality, generality, and uniformity in our word choice: we write about a general group of *people (aggregate level)*, not a specific type of *person (individual level)*.

A shift from nomothetic to a group-specific level appears as an attempt to come closer to the person. However, the closer the Cartesian-split-mechanistic research agenda wants to come to the person, the more it leaves its comfort zone and open itself to critics. How can a primarily population-oriented and variable-oriented methodology grasp the particularities of a singular person? It seems out of reach. Consequently, a standpoint “specialized” in the analysis of the person is required as a complement.

### Mapping the road 2: Bottom-up to the type-specificity, but not farther

Inversely, the processual-relational worldview starts the developmental analysis from the bottom-up—from the particular to the general—by keeping idiographic focus as long as possible. Person-oriented researchers believe interaction—not variables—are relevant. Thus, what “distinguishes a person from other persons is the patterning of these various aspects of the individual functioning as a totality” (Magnusson, 1988, p. 47).

**Figure 7:**
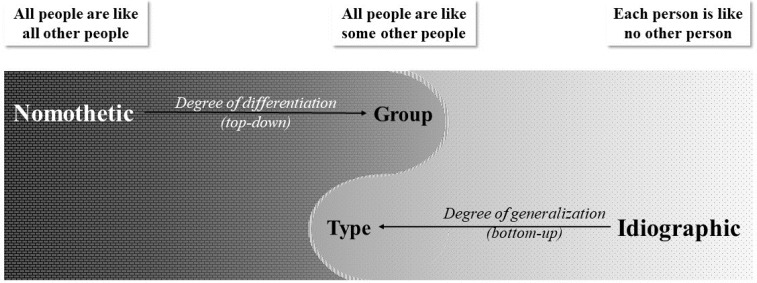
Revisiting the three kinds of laws.

However, researchers in the human sciences, including talent research, typically acknowledge that science cannot be reduced to the sole description of the uniqueness of an individual functioning as a totality (Magnusson, 1988). Thus, researchers aim to discover what is general or common in addition to what is particular, unique, or idiographic—implying some kind of generalization, such as aiming for group-specific statements. In this context, group-specificity is achieved by identifying similar *types* or patterns of individuals functioning across persons. Researchers use clustering algorithms to find different *types* of persons and create a prediction model for each type (Bergman et al., 2003). This allows them to make broader statements that go beyond individual cases while still maintaining a focus on understanding specific individuals, thereby staying close to the idiographic pole. Consequently, and for precision’s sake, we recommend the words *type-specificity* rather than *group-specificity* in the context of bottom-up analysis (see Figure 7 for visual representation). Since different types of persons have different success probabilities (Zuber et al., 2016), knowing the ‘type’ affiliation allows for interindividual comparisons and individual evaluations. Please note our idiographic idea of singularity in our careful, purposeful word choice: we write about a specific type of *person*, not a general group of *people*.

Shifting from idiographic to a type-specific level is an attempt to move closer to the population. However, the closer the processual-relational research agenda wants to come to the population, the more it leaves its comfort zone and opens itself to criticism. How can a primarily specificity-oriented methodology grasp the generalities of a population? It seems out of reach. Consequently, a standpoint “specialized” in analyzing populations is required as a complement.

### Pragmatic coordination of the two standpoints

**Figure 8:**
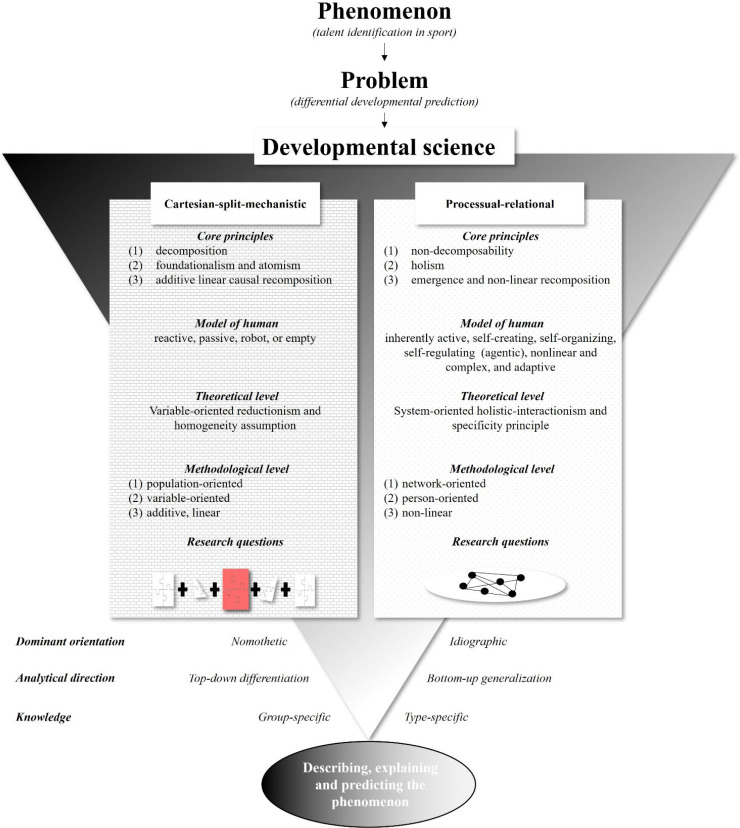
Talent identification research: a (meta-)theoretical and methodological overview.

To sum up, we have two aspects that continue to fall short: (1) in its quest for nomothetic (or group-specific) knowledge, scientific support from the Cartesian-split-mechanistic worldview fails to adequately consider the particularities of a singular person; (2) in its quest for idiographic (or typespecific) knowledge, scientific support from the processualrelational worldview falls short in grasping the generalities of a population. Taken together, we propose to enhance talent identification by coordinating decision-making tools from both worldviews (see Figure 8).

From a practical perspective, it is essential to acknowledge the valuable insights that scientific support from both worldviews can provide, especially when confronted with the inherent limitations of human intuition in predicting developmental outcomes (e.g., Grove, 2005; Sieghartsleitner et al., 2019). This raises two fundamental questions: (1) When should the scientific support occur? Should the human initially assess potential, feeding the findings into the scientific algorithm, or should the algorithm make a prediction for the human to consider afterward? (2) How could we coordinate the worldviews?

Concerning the first question, in alignment with previous perspectives (Den Hartigh, Niessen, et al., 2018; Hecksteden et al., 2023; Woods et al., 2021), we recommend utilizing scientific knowledge and algorithms to augment and extend human intuition rather than overwriting and replacing it. Given that selection decisions profoundly impact an individual’s future, an ethical approach to talent identification should support choice and agency in the matter. Indeed, there is consensus in talent research that the final decision should be a human one rather than dictated by algorithms (Lath et al., 2021; Sieghartsleitner et al., 2019).

Concerning the second question, we suggest four steps to coordinate both worldviews, transitioning from a Cartesiansplit-mechanistic worldview to a processual-relational one and from a nomothetic-oriented approach to an idiographicoriented one. This proposal is a starting point, not an exhaustive conclusion. Thus, these four steps may be subject to adjustment based on evolving insights. The four steps are as follows: (1) acknowledge the relevance of talent criteria; (2) utilize algorithmic predictions; (3) incorporate guidance from person-oriented studies to refine intuition; and (4) consider the whole person.

1. To discern and become acquainted with the “shopping list of criteria” (Williams & Reilly, 2000, p. 658), the model of human as machine is appropriate (see Figure 3). As a starting point, it is necessary to spotlight key pieces of the talent machine (talent predictors) (Höner et al., 2021) as well as the ones possibly leading to selection errors (Johnston & Baker, 2020) or the ones representing no-go’s with almost no chance of future success, such as predicted adult height of 1.5 meters for volleyball.
2. Considering insights from decision-making literature (Grove et al., 2000; Kahneman et al., 2021) and talent identification research (Sieghartsleitner, Zuber, Zibung, & Conzelmann, 2019; Siener et al., 2021), it becomes evident that the talent identification process should integrate the results of prediction models. This includes success probabilities derived from nonlinear algorithms, additive-linear models, or a combination of both, in conjunction with a thorough analysis of raw data.
3. Coaches who make final decisions need to finetune their intuition. Fine-tuning human intuition becomes challenging if scientific support reduces to showing coaches a number on a scale (e.g., predicted success probabilities as total score). It appears that guidance for fine-tuning human intuition which is *loyal* to the nature of the phenomenon and *transparent*, may only arise from the insights derived through person-oriented analysis. Here, coaches can learn to recognize the context- and time-dependent good *Gestalt*, such as the type of person with (sub-)optimal developmental path (see Figure 6), based on psychological (e.g., see Zuber et al., 2015), sociological (e.g., see Lenze et al., 2023), training (e.g., see Sieghartsleitner et al., 2018) or motor profile (e.g., see Zibung et al., 2016).
4. Armed with knowledge from the previous steps as well as with a delayed, fine-tuned, and thus person-oriented intuition, coaches can proceed to the fourth and pivotal step: considering the whole person in the act of the selection decision. Coaches describe this precise moment as “one of the most challenging aspects of their job” (Neely et al., 2016, p. 141). At this moment, we recommend them to engage in processual-relational thinking (for defining features, see Lerner, 2007), considering the entire life story of the self-creating, self-organizing, agentic, complex, and adaptive individual, possibly encompassing variables not explicitly addressed in the initial steps and previous knowledge about exceptional single case studies (e.g., John & Thiel, 2022a). In doing so, coaches construct a truly dialectical, holistic, and developmental picture in their minds, one that transcends the mere sum of its parts and informs their decision to select the individual or not.

To conclude, we return to the paper's title: mechanistic *or* relational worldview for talent identification research in sport science? After thorough theoretical and applied considerations, our answer is *both*: Talent identification requires both mechanistic and relational knowledge at different steps of the process. However, when considering the fidelity to the phenomenon, a preference for the relational worldview appears logical, especially when transitioning from talent identification to the practical decision-making process of selecting the *individual*.

## Data Availability

Data sharing is not applicable to this article as no datasets were generated or analyzed during the current study.

## Action editor

Lars-Gunnar Lundh served as action editor for this article.

## Open access

This article is distributed under the terms of the Creative Commons Attribution 4.0 International License (http://creativecommons.org/licenses/by/4.0/), which permits unrestricted use, distribution, and reproduction in any medium, provided you give appropriate credit to the original author(s) and the source, provide a link to the Creative Commons license, and indicate if changes were made.
